# Artificial Intelligence in Informal Digital Learning of English: A Systematic Review of Engagement Mechanisms and Learning Processes in Intelligent Learning Systems

**DOI:** 10.3390/jintelligence14080189

**Published:** 2026-08-18

**Authors:** Lihang Guan, Qingbo Yang

**Affiliations:** School of Foreign Languages, Ludong University, Yantai 264000, China; lguan@ldu.edu.cn

**Keywords:** AI-mediated informal digital learning of English, adaptation of intelligence in self-regulated learning, social cognitive theory, technological affordances, learning engagement

## Abstract

Artificial intelligence (AI) creates personalized, interactive, and low-risk learning opportunities for informal digital learning of English (IDLE). However, effective AI tools do not necessarily lead learners to engage autonomously or productively. Guided by the person–behavior–environment framework of social cognitive theory, this systematic review synthesized 58 empirical studies published in SSCI-indexed journals between 2020 and 2025 to examine how AI affordances are translated into learning behaviors and intelligence-related processes in AI-mediated IDLE. Speech recognition, natural language processing, and personalized feedback were the most frequently investigated affordances, providing opportunities to observe, imitate, and adapt authentic language use. Engagement was mediated primarily by emotional, motivational, and self-belief mechanisms. These mechanisms could facilitate or hinder engagement depending on technological accuracy, perceived human responsiveness, learner goals, and proficiency-task alignment. From an intelligence perspective, the findings illustrate how learners evaluate AI-generated information, regulate affect and motivation, calibrate self-perceptions, and adapt learning behaviors. Thus, AI-mediated IDLE may serve as a site for activating adaptive intelligence and learner self-regulation through context-sensitive evaluation, regulation, and behavioral adaptation.

## 1. Introduction

The integration of artificial intelligence (AI) into English as a foreign language (EFL) learning has reshaped traditional teaching and learning methods. With unique technological affordances like natural language processing, immediate and personalized feedback, and multimodal outputs, AI technologies offer new experiences for personalized learning, efficient assessment, and increased learner-centered EFL learning opportunities (Xi et al., 2026). For many researchers (e.g., Xi et al., 2026), the significance of such opportunities lies in the transformation from teacher-led instruction-based English learning to personalized, learner-centered English learning. Through the customization of learning experiences in particular, AI technologies have been found to analyze individual learners’ input, providing immediate and tailored feedback and thus generating content more suitable for learners’ individual learning needs (Yeh, 2024). In this learner–AI collaboration, not only are learning contents co-constructed between learners and AI for learning personalization (Guan et al., 2025a), but also AI enables learners to thrive through opportunities to observe and imitate authentic language outputs scaffolded with immediate and catered assistance (Guan et al., 2025b; Shen et al., 2025).

Studies have also found, however, that giving learners AI-mediated learning opportunities for personalized observation and imitation does not guarantee EFL learning (Guan & Gu, 2026). Ssocial cognitive theory (SCT; Bandura, 2014) has long suggested that, although observation and imitation are crucial to language mastery, the effects of such learning behaviors are contingent on the reciprocal system among personal, behavioral, and environmental factors. In other words, in order for learners in specific learning environments to demonstrate such effective observation and imitation behavior, agency needs to be exercised (Guan et al., 2025c). With specific reference to the AI-mediated informal digital learning of English (AI-IDLE), in which engagement (e.g., focus on form or focus on meaning) has always been a learning-quality concern (Lee, 2019), an investigation of the mechanisms driving learners to effective observational and imitative behaviors was thus warranted.

With this understanding, this systematic review synthesizes the existing literature to explore how AI technological affordances—understood as the action possibilities that AI tools offer to learners in informal digital environments (e.g., speech recognition, personalized feedback)—encourage learners to engage autonomously in AI-IDLE guided by the person–behavior–environment triad, which explains how learning actions are negotiated within the SCT framework (Bandura, 2014). This review makes both theoretical and practical contributions. Theoretically, it applies SCT, an influential cross-disciplinary theory of human functioning that has been adapted into language-learning research (e.g., Guan et al., 2025b), to explain how AI affordances shape engagement through learners’ emotions, motivation, and self-beliefs. It also connects AI-IDLE with adaptive intelligence, defined here as learners’ context-sensitive capacity to evaluate AI output, regulate internal states, and adjust learning behavior. Because the included studies rarely measured intelligence directly, this connection is interpretive rather than evidence of changes in general intelligence. Practically, the review informs human-centered AI design, learner autonomy, and AI literacy.

## 2. Literature Review

### 2.1. AI-IDLE and AI Affordances

IDLE has been conceptualized as learners’ autonomous English learning activities mediated by digital technologies, normally in forms of feedback and collaboration, with or without a relationship to formal education (e.g., a curriculum or textbook) (Lee & Dressman, 2018). However, the gray areas defining formal and informal learning (Guan et al., 2025b) dictate a more flexible consideration. This study does not adopt the strictest definition of IDLE which confines informal learning to out-of-class, fully self-taught and fully self-controlled activities with no relevance to in-class education, but evaluates the literature based on whether students have autonomy to self-direct their learner–technology interactions. This conceptualization can reach a broader range of the literature that grants certain degrees of autonomy, instead of fully controlled step-by-step guidance to students for a more generalizable interpretation of human–computer interactions.

AI-IDLE is, therefore, conceptualized as a set of English learning activities with certain levels of learner autonomy in the interactions mediated by various AI technologies, from AI-integrated dictionaries to chatbots (G. L. Liu et al., 2025a). AI-IDLE mediates contexts of self-directed learning activities in human–computer interactions in addition to non-AI providing access to authentic English uses with technological means (Guan et al., 2025c; G. L. Liu et al., 2025a).

These differences in technological affordances and adaptation bring distinctively different digital learning patterns that can shape the SLA process and outcomes with new learning paths. First, AI tools may offer personalized feedback as learning inputs and outputs for learners’ language-skill development (G. L. Liu & Zhao, 2025). Studies have found that AI can quickly identify learners’ grammatical errors and inappropriate vocabulary usage in writing or speaking outputs (Shen et al., 2025) and give targeted modification suggestions that are compatible with their level of language proficiency (Shafiee Rad, 2024). Second, AI could construct adaptive content recommendations, which support and stimulate two-way learner–technology interaction, allowing them to co-create learning content in authentic learning contexts (e.g., ordering food at a restaurant). This co-construction not only negotiates ongoing attention to both contextual meaning and linguistic form, a combination that underpins IDLE’s effectiveness (Guan & Gu, 2026; Lee, 2019), but also provides continuous opportunities for learners to observe and imitate language uses in content negotiations, further improving their understanding of authentic language use in different communicative processes and scenarios. Unlike the relatively static materials provided by traditional, non-conversational IDLE technologies, AI, especially in its generative forms, can offer real-time dialogue tailored to specific learning scenarios. Such interactions may improve learners’ cognitive, affective, and behavioral engagement under SCT, which explains human functioning through interactions among personal factors, behavior, and the environment.

Therefore, unlike pre-designed static learning materials on traditional, non-conversational IDLE technologies, AI, especially in its generative form, offers additional real-time dialogues specific to the learning scenarios, improving both the quantity and quality of learners’ cognitive, affective, and behavioral EFL learning engagement under the SCT framework (a theoretical perspective viewing learning as a socially mediated human-functioning process manifested in the interactions among personal factors, behavior, and the environment) (see Section 2.2 for a complete definition).

AI also has the potential to create simulated learning environments that create virtual learning conditions in which a learner collaborates or competes with simulated agents (e.g., virtual teammates and competitors) in human-like interactions (Hu & Chan, 2025). In empirical studies, the adaptive use of these immersive simulations was found to help learners lower their communicative anxiety and boost their speaking self-efficacy, making them more willing and capable when communicating with real people in social scenarios (Biju et al., 2024; Fathi et al., 2024). AI-simulated learning environments, then, function as low-risk spaces for practice, allowing learners to polish their English communication skills repeatedly (Isabella et al., 2026). Different from traditional out-of-class digital English learning, which relies mostly on one-way information reception, the simulated environment aligns more closely with the SLA processes and modern SLA theories, thereby enabling learners to accumulate human-like communicative experiences outside formal classrooms.

Current research on AI affordances in AI-IDLE, however, still has notable gaps, especially in its mechanisms. On the one hand, most existing studies have focused on the effects of AI applications in formal English classrooms (e.g., Jeon & Lee, 2024), while fewer empirical studies have been conducted on the effectiveness of AI-IDLE. It is true that the autonomous nature of AI-IDLE (Guan & Gu, 2026; Lee, 2019) challenges the possibility of doing interventions and explorations within IDLE’s definition. Considering, however, the extracurricular and extramural categories of IDLE (which are classified according to IDLE’s proximity to formal education) research actions in the form of flipped classrooms and interest clubs could be executed within the IDLE framework (e.g., Huang et al., 2023; Jeon & Lee, 2024; Q. Zhou et al., 2025). This would leave room for further IDLE experimental studies. On the other hand, the existing explorations of AI affordances often only describe the technical functions of the tools themselves (Ng, 2022). They also often lack in-depth analysis of how these technical possibilities interact with learners’ personal characteristics for the deep learning engagements with cognitive processing that ultimately produce learning effects (Fathi & Rahimi, 2024). Therefore, by synthesizing those AI-IDLE studies that have reported technological affordances and learner interpretations, this study also provides theoretical and practical pathways for IDLE and AI-IDLE experimental designs under the SCT framework.

### 2.2. Social Cognitive Theory

Various SLA theories underline the significance of the psychological processing of input and output in language development. The cognitivist perspective dictates that authentic language input must be noticed (e.g., the information processing theory of Segalowitz, 2003), meaningfully connected (e.g., connectionism by Ellis, 2003), monitored (e.g., the monitor model of Krashen, 1982), and applied (e.g., the output hypothesis of Swain, 2000). Although Bandura’s social cognitive theory (SCT) was developed as a cross-disciplinary theory of human actions and interactions (Bandura, 2001), it has also been applied to language-learning research, particularly in studies on language-learning self-efficacy, motivation, and strategy use (e.g., Guan et al., 2025b). From the SCT perspective (Bandura, 2014), language learning is viewed as a form of observational learning in which learners notice, interpret, and model target-language behaviors in social and mediated environments. Some even argue that such observational learning is a strategy that connects individual learner beliefs to learning attitudes and actions (Zhu et al., 2026; Ye & Hu, 2026).

Following the conventions of applying SCT to SLA (e.g., Guan et al., 2025b; Zhu et al., 2026; Ye & Hu, 2026), we use observation as a shorthand for processes like noticing, making form-meaning connections, and monitoring input, and imitation as a shorthand for learners’ attempts to reproduce, adapt, and experiment with linguistic patterns. We acknowledge that language acquisition also involves more complex processes (e.g., hypothesis testing, creative language use, metalinguistic reflection), but we adopt this observation–imitation framing as a heuristic to organize different types of engagement in AI-IDLE.

Therefore, SCT provides a suitable lens through which to analyze how AI affordances translate into actual learning behaviors through learners’ situated responses in AI-IDLE. As discussed in Section 2.1, AI-IDLE differs from traditional IDLE in its ability to mediate practice, provide feedback, and participate in content co-construction (Guan et al., 2025c); thus, it can create technologically oriented learning environments that are stimulative, interactive, and engaging for learners to enact within. Therefore, from the SCT perspective, AI affordances can be understood as environmental catalysts that shape learners’ personal factors, such as self-efficacy, anxiety, and motivation, thus provoking different internal individual characteristics that influence how they interpret and respond to the learning environment, leading to observable actions and patterns of actions known as behavioral engagement (Bandura, 2014; Guan et al., 2025b). This understanding not only aligns with SCT’s emphasis on person–behavior–environment interplay in education but also situates the investigative factors of learner interpretation in mechanisms specifically in the environment–person–behavior logic, with AI-mediated technological environments as the situational context, learners as the main body of actions, and learning behaviors as the product.

Previous studies have found that the technological environment can optimize learning engagement through exerting influences on individual learners (e.g., Hu & Chan, 2025). Specific to the EFL context, studies found that those learners who interact with AI chatbots not only received more practice opportunities in the form of input and output but also experienced affective values, such as reduced anxiety and enhanced enjoyment, both of which together improved language proficiency (e.g., Guan et al., 2025b; Teng, 2024). From the perspective of AI technology, natural language processing abilities allow learners to receive authentic and accurate language exchanges in which they actively communicate and correct language production within learner–AI interactions (G. L. Liu et al., 2025b; X. Wang et al., 2023). Moreover, the multimodal outputs of AI also support the formation of meaningful connections that stimulate learners’ cognitive engagement (Shahipanah et al., 2025). More directly linked to SCT, some studies have found that receiving immediate and personalized feedback from AI helps learners imitate the observed linguistic segments in context-sensitive environments, leading to deeper cognitive engagement, such as a dual focus on contextual form and linguistic meaning (Lee, 2019; Mohamed, 2024). Therefore, SCT’s emphasis on the reciprocal triad constructs a suitable and empirically validated framework for synthesizing AI affordances and AI-related mechanisms in motivating autonomous engagements for learners.

Based on the previous review of the literature, we propose that AI technological affordances, as well as their interpretations, can be better examined through the person–behavior–environment triad in SCT, which situates learning decisions and behaviors in a dynamic interplay of influences both within each learner and the learning context. In particular, the environmental factor in SCT marks the technological environment and explains how AI provides authentic language exposure for observation and imitation (opportunities for learning). The personal factor pertains to the individual differences of learners, which explains how these AI affordances are transformed by learners into actions (mechanisms for learning). Within specific combinations, the environmental and personal factors may be interpreted into frequent and deep engagements, where learning happens (Bandura, 2014). For these investigative purposes, we propose the following research questions:

(1) What technological affordances in AI-IDLE have been adopted to provide observation and imitation opportunities for English learning?
(2) Through what person-level mechanisms do these affordances facilitate or hinder learners’ observation and imitation behaviors in AI-IDLE?

## 3. Methodology

### 3.1. Literature Search

This systematic review followed the PRISMA 2020 guidelines (Page et al., 2021) and was registered in PROSPERO (#1381535). For a comprehensive overview of the literature, we searched for publications published from 2020 to 2025 in all major databases, including Web of Science, Scopus, ProQuest, and Google Scholar, with the following keywords and after discussions between both authors:

(“informal” OR “out-of-class” OR “after class” OR “beyond classrooms” OR “autonom*”) AND (“intelligent” OR “AI” OR “artificial intelligence” OR “IPA” OR “ChatGPT” OR “chatbot” OR “Google Assistant”) AND (“English as a foreign language” OR “EFL” OR “language learning” OR “language teaching” OR “L2” OR “language education” OR “second language acquisition”) AND (“empirical” OR “experimental” OR “quasi-experimental” OR “quantitative” OR “qualitative” OR “mixed*”)

Because Google Scholar does not support complex Boolean queries, we selected representative search keywords, (“autonom*”) AND (“artificial intelligence”) AND (“English as a * language”) AND (“*empiric*”) and reviewed the first 50 pages of results. To maximize the inclusion of articles, we also conducted backward citation tracking from previous AI-in-EFL-related systematic reviews (e.g., Y. Liu et al., 2024). Together, these databases yielded 6419 articles.

### 3.2. Data Inclusion and Exclusion Process

**Figure 1:**
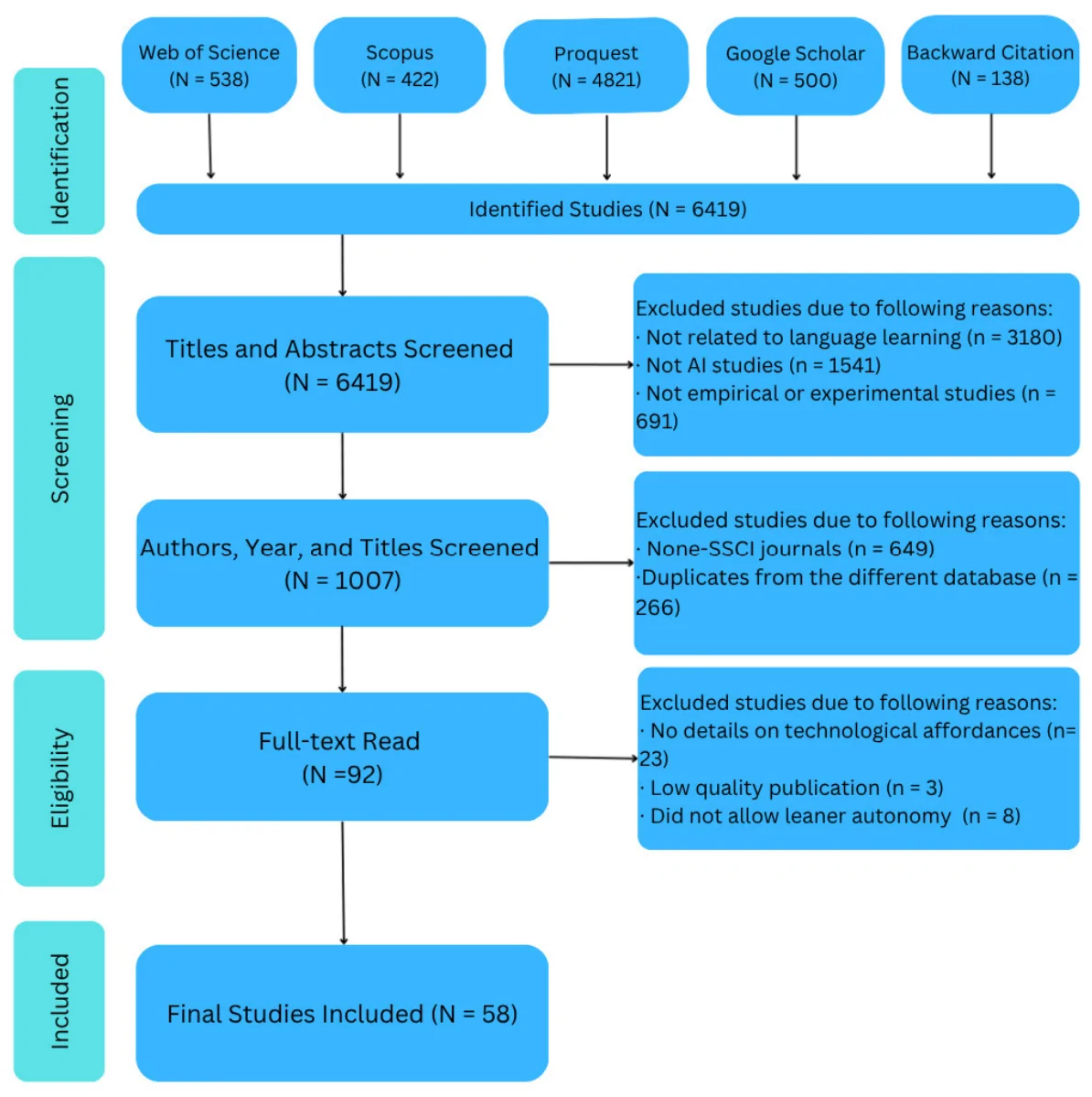
PRISMA flow diagram.

We developed explicit criteria to identify peer-reviewed empirical studies reporting AI affordances and learner-related mechanisms in AI-IDLE. Title and abstract screening excluded records unrelated to informal English learning (*n* = 3180), AI-based learning (*n* = 1541), or empirical research (*n* = 691). We then excluded non-SSCI publications (*n* = 649) and duplicates (*n* = 266). Full-text screening of the remaining 92 studies excluded 23 that inadequately described AI use, three with insufficient methodological or empirical reporting, and eight that provided little learner autonomy. The final sample comprised 58 studies. We also set an exclusion criterion to exclude formal learning studies, following the conceptualization by seminal works (Lee, 2019; Lee & Dressman, 2018) of informal learning—autonomous out-of-class learning that may be connected or unrelated to formal classroom content. We found no formal learning studies among the remaining studies. The selection process is illustrated in Figure 1. The Supplementary Material provides the references for all included studies.

### 3.3. Data Analysis

Both authors independently extracted full-sentence excerpts from the 58 studies for thematic analysis. The unit of analysis was full-sentence excerpts that explicitly described: (1) AI technological affordances used to provide EFL learning opportunities and/or (2) how individual learners respond to these opportunities. Codes were not mutually exclusive; a single excerpt could receive multiple codes. All frequency counts (*n*) refer to the number of studies in which a given code appeared at least once, rather than to the raw number of coded excerpts. NVivo 20 was used for deductive coding and thematic analysis, following the step-by-step guide of Braun and Clarke (2006) theoretically informed by SCT. In the data-familiarization step, we carefully read all the included studies to become familiar with their content. To generate the initial codes, we then highlighted each segment presenting information related to environmental and personal factors in the included articles before incorporating them as labeled excerpts in a Google online document. In the thematic-identification step, the excerpts were re-examined within the original publications to ensure accuracy and complete understanding. Lastly, these excerpts were sorted into themes and subthemes. After an initial round of independent coding of all 58 studies, we calculated inter-coder reliability between the two authors using Krippendorff’s alpha, which reached 0.92, and disagreements were resolved through discussions and re-examinations of the excerpts within the context of the original studies. Examples of the coding and resulting themes are provided in the codebook in the Supplementary Materials.

## 4. Findings and Discussion

### 4.1. Technological Affordances by AI-IDLE to Provide Observation and Imitation Opportunities for English Learning

The thematic analysis identified the most common affordances in AI-IDLE: AI’s speech recognition (*n* = 30), natural language processing (*n* = 30), and personalized feedback (*n* = 17). These affordances, used either in combination or separately, provided EFL learners with multiple learning opportunities to observe and imitate authentic language inputs and outputs. These opportunities often take the form of communicative interactions (Guan et al., 2025b) and corrective revisions (Zhang et al., 2024). For example, in a study by Zou et al. (2024), learners interacted with the application EAP Talk, where they received not only responses from the AI chatbot but also suggestions for linguistic revisions of their real-time communications, which “allowed students to engage in extensive speaking practice independently” (p. 8).

Regarding individual language competence, these technological functions were used mostly in speaking (*n* = 30), making it the most frequently studied language competence in AI-IDLE. The other language competences with AI-IDLE activities—writing (*n* = 14), reading (*n* = 3), and listening (*n* = 2)—were explored much less frequently than speaking. Moreover, despite researchers’ favoritism of studying language competence developments in AI-IDLE, the use of AI technological affordances and the modal processing of the language competences are similar in modality adaptation, with a prevalence in adapting the oral modality (*n* = 41), even in reading and writing studies. For example, in a study by C. C. Liu et al. (2022) on EFL reading, learners were asked to speak with SisterFish (an AI agent) about the content of the book they had just read to enhance their comprehension, indicating their use of AI’s speech recognition and natural language processing affordances as if in speaking-oriented AI-IDLE activities. Similarly, the study of C. Zhou and Hou (2024) found that learners used AI’s speech recognition and translation affordances to help them understand listening passages through oral consultations with AI. Therefore, the prevailing paradigms in the reviewed studies of commonly researched language competences and the use of AI affordances correspond to the study of Soyoof et al. (2023), although the adaptation of technological modalities in IDLE remains underexplored.

### 4.2. Personal Factors as Mechanisms for Observation and Imitation Behaviors in AI-IDLE

For the interpretations of AI-IDLE affordances, we identified three main themes—emotional, motivational, and self-belief mechanisms—each of which contains different numbers of subthemes. Combined, they depict a holistic view of how AI drives engagements in learner–AI interactions.

#### 4.2.1. Emotional Mechanisms of AI-IDLE

Bringing emotional value to learning is one of the most frequently examined mechanisms for promoting learners’ (*n* = 52) engagement. Within the examined emotions, the most common subtheme in AI-IDLE is reduced anxiety (*n* = 42). Studies found that “the experimental group—who underwent GenAI-assisted language assessments—showed a clear drop in anxiety levels” (Biju et al., 2024, p. 11), mostly because of no sense of judgment from others (Jeon & Lee, 2024). Compared with this strong consensus, other emotion-related interpretations in AI-IDLE were much less frequently discovered. The thematic analysis also identified enjoyment (*n* = 15), comfort and relaxation (*n* = 10), finding the activities fun and interesting (*n* = 10), and novelty (*n* = 5), but anxiety was mentioned more than four times as often as any of these. Regardless of the uneven distribution of emotional benefits, researchers have argued that most of the emotional engagement mechanisms originate in the non-judgmental environment provided by AI-IDLE. A study by Tai and Chen (2024b) perfectly reflects how the elimination of judgment can lead to reduced anxiety, enjoyment in learning, feelings of comfort and relaxation, and having fun in the learning process. Their study (Tai & Chen, 2024b) reported that learners’ interactions with the Alexa device “added [learning] variety and enjoyment to EFL speaking” (p. 1224), making them “more comfortable and confident with speaking English” (p. 1238), and “sparked the GA-Hub group’s interest” because the “interactions yielded less anxiety compared with face-to-face interactions” (p. 1239). Therefore, we believe that reduced anxiety may be the most significant emotional value of AI-IDLE for learners since many reviewed studies reported that their participants believed human partners to be more judgmental than AI partners.

There are, however, also negative experiences found in AI-IDLE regarding the emotional mechanisms. The main challenges were technological (*n* = 10) and pedagogical (*n* = 10), hindering the interpretation from mechanism to action. Among the technological challenges, AI’s mistakes in speech recognition and generated output dominated the negative experiences EFL learners were reported to have with AI-IDLE. In a study that used multiple AI systems to foster learners’ speaking proficiency (Zou et al., 2023), more than half of the participants (undergraduates) felt unpleasant when AI failed to record their language competence improvements. Therefore, although AI technologies have been found to reduce anxiety and improve users’ willingness to communicate (e.g., Fathi et al., 2024), the performance of current AI systems is imperfect. This imperfection creates negative emotions in learners engaging in AI-IDLE, which can hinder the effectiveness of personal factors as mechanisms that drive autonomous observation and imitation in AI-IDLE.

The pedagogical challenges learners face in AI-IDLE appear mostly to reside in extracurricular IDLE activities: those IDLE activities unrelated to formal education (Lee, 2019). For example, Guan et al. (2025b) found that, although learners deemed AI agents to be highly useful for their speaking proficiency, they struggled to maintain the practice because of their “lack of enjoyment” (p. 11) in learner–AI interactions. C. Wang et al. (2024) explored this lack of enjoyment and suggested that AI “doesn’t evoke the same deep emotional response” (p. 11) as an experienced teacher. The same was found in Teng’s (2024) study of AI-IDLE writing experiments, in which participants claimed that AI lacked the “human touch and emotional understanding that a human evaluator or peer might provide” (p. 8). This lack of human touch may also hinder the most significant emotional value brought to learner–AI interactions: the reduction in anxiety, which strengthens self-regulation in informal learning (Edwards et al., 2025). As one participant in Rahman and Tomy’s (2024) study said, “I don’t feel anxious when I converse with AI. However, the moment I talk to people, I get anxious again” (p. 4579). Of the reviewed studies, 27 (about 47%) found that the lack of a human touch contributes to emotional disengagement in AI-IDLE. The high number of insufficiencies in current AI technologies suggests that supplements to the technological environment may be needed to make AI effective within autonomous digital EFL learning. In AI-IDLE, one promising avenue to making AI more effective in pedagogy could be learner-initiated collaborative informal digital learning: for example, using chatbots to brainstorm and generate collaborative speeches such as those in an English debate.

#### 4.2.2. Motivational Mechanisms of AI-IDLE

Thematic analysis found that EFL learners were driven toward deep engagement in AI-IDLE because they found the AI technology useful (*n* = 21) and convenient (*n* = 12) and because it provided additional practice opportunities (*n* = 11), autonomy (*n* = 11), human-like interactions (*n* = 6), and improvements in language production quality (*n* = 4). These motivational benefits are not as inherently consistent as the emotional benefits among all EFL learners; rather, they seem to be dependent on individual differences. Learners with upcoming tests (e.g., final term exams, standardized English tests) appear to turn AI-provided additional learning into action opportunities more strongly than others. For example, in a study by Guan et al. (2025b), learners preparing to take tests of the International English Language Testing System appreciated having additional communicative opportunities with generative AI to observe and imitate AI’s authentic language usage before taking the tests. After taking the test, however, they quickly abandoned these observational and imitative behaviors (Guan et al., 2025b). On the other hand, when learners have no upcoming important tests, the convenience and autonomy of AI-IDLE may be more valuable for learning motivation and engagement (Guan & Gu, 2026). Participants in a study by Chen et al. (2023) reported that they “believed that [the AI agent] could boost their motivation in learning English pronunciation, vocabulary, listening, and speaking” (p. 1342) when they “self-repeat[ed] their problematic utterances more often” (p. 1347) in self-regulated learning. Therefore, there could be distinguishable differences in learners’ motivational mechanisms that drive learning engagements in AI-IDLE that are dependent on external pressures and individual differences. Learners’ goals and time-bound external pressures may also reshape how the same AI affordances are taken up, perceived, and sustained over time, modifying the functional learning environment created by AI-IDLE. The influences of the person on the environment in the reciprocal person–behavior–environment triad are less explored, leaving little evidence in this area for this review to synthesize.

The motivational mechanism also presents its own challenges, which have been found to involve the same categories as the emotional challenges: technological (*n* = 19) and pedagogical (*n* = 16) difficulties. For the technological and pedagogical difficulties in motivational-mechanism interpretation, some studies have argued that learners do not want to use AI in their EFL learning because AI “ha[s] problems accurately recognizing accented speech” (Tai & Chen, 2024a, p. 1240), “produc[es] inaccurate writing samples” (Fathi & Rahimi, 2024, p. 19), and “[can’t] replace the humanistic aspects of teaching and learning” (C. Zhou & Hou, 2024, p. 10) because the current AI tools cannot accurately “interpret human emotions and implicit meanings” (C. Zhou & Hou, 2024, p. 8). Thus, studies have suggested that “most students continue to view AI tools as incapable of fully replacing human instructors” (Y. Wang, 2024, pp. 8–9) and that they regard them “as a complement to human educators” (Y. Wang, 2024, p. 9), which supports the presence of human involvement in intelligent learning systems in broader educational contexts (Cukurova, 2025).

Comparing these technological and pedagogical challenges with the ones in the emotional theme, we can identify several similarities. First, similar technological issues and pedagogical concerns were raised by learners regarding both the emotional and motivational mechanisms. Factors like errors in speech recognition and the lack of a human touch hinder both EFL learners’ emotional experience and their motivation to engage deeply in AI-IDLE. According to SCT (Bandura, 2014), individuals’ physiological and emotional states in the interactive learning experience affect their ability to manage stress, anxiety, or fatigue, which strongly affects one’s willingness to engage in language-learning activities. In other words, learners’ motivation to engage deeply in AI-IDLE can be closely connected to their experiences with AI technologies. Since some learners face technological and pedagogical difficulties in perceiving positive emotional and motivational values, these issues must be addressed. As argued by multiple studies (e.g., Bardach et al., 2026; Cukurova, 2025; Tian & Xiang, 2026), possible research areas in which to address these issues include AI literacy, human agency, and scaffolded intelligent system designs.

#### 4.2.3. Self-Belief Mechanisms in AI-IDLE

In the included studies, self-confidence typically referred to learners’ general beliefs that they could accomplish communicative tasks, while self-efficacy and self-perceived communicative competence referred more specifically to beliefs about successfully conveying messages and being understood in interaction (e.g., Teng, 2024; Y. Wang, 2024). Reports of being able to understand AI-generated language were therefore coded as self-efficacy/self-perceived communicative competence, whereas reports of feeling less nervous when interacting with AI instead of humans were coded as lower anxiety and discussed under emotional mechanisms.

This self-belief mechanism in AI-IDLE in the reviewed studies mostly took the form of self-perceived competence (*n* = 17), with most learners believing in their own language competences, both current (*n* = 9) and developmental (*n* = 10). Regarding perceived current competence, the reviewed studies found that learners showed higher self-belief in learner–AI interactions, as they showed “little worry about not being understood by GenAI due to their poor English” (Zhang et al., 2024, p. 9) and “more confidence in getting their message across without feeling nervous.” Concerning perceived developmental language competence, the studies suggest that AI-IDLE “promotes user agency to build confidence gradually” (Shafiee Rad, 2024, p. 366) by providing “a low-stakes, non-judgmental environment for practice” (p. 375). Therefore, self-perceived language competence is a subjective appraisal of communicative ability and should be distinguished from objectively assessed proficiency. By providing a safer learning environment, AI may reduce anxiety and strengthen learners’ perceived communicative competence, thereby encouraging engagement and sustained practice. Such engagement may, in turn, support language proficiency development (C. Zhou & Hou, 2024). AI may therefore create a positive engagement cycle between perceived competence and continued language practice.

The self-belief mechanisms are not without their own challenges. In AI-IDLE engagements, such challenges come mainly from the mismatch between learners’ English proficiency levels and AI’s language usage (*n* = 14). In most of these studies’ reported challenges, the participants had insufficient language proficiency to comprehend the authentic language generated by AI technologies. For example, 43% of participants in Tai and Chen’s (2024a) study reported that they had difficulty understanding the communicative responses of the chatbot. Although this phenomenon may be attributed to the young age of the participants (sixth graders) and their limited experience with English (Tai & Chen, 2024a), similar difficulties have also been observed among college students (e.g., Zhang et al., 2024), meaning this phenomenon might be common among low-proficiency EFL learners of all ages. This finding is contrary to the findings of some studies (e.g., Shafiee Rad, 2024; Yuan, 2024) that have suggested that AI feedback is easier to understand than teachers’ feedback because of the multimodal outputs (e.g., audio and written) that AI generates. In fact, Tai and Chen (2024a), among others (e.g., Zhang et al., 2024), have recommended the use of both audio and written formats in learner–AI interactions, but in 13 of the included studies, some learners still struggled to understand and sustain learner–AI communication. Examining Hsu et al.’s (2023) study, which clearly stated that “the learners could understand Alexa” (p. 5739), we discovered that it used gamification elements in collaborative contexts. Thus, it is possible that AI’s technological affordances need to be organically designed into a social learning environment for effectiveness.

Notably, English proficiency appears to hinder learner engagement in AI-IDLE not only for low-proficiency learners but also among advanced learners. The findings of Tai (2024) suggest that AI’s communicative responses are “not challenging for these [advanced] learners. Therefore, there is little room for improvement” (Tai, 2024, p. 1302). This leads to lower levels of engagement in such interactions. We do not question that AI can be adaptive to learners’ needs when used properly, but we suggest that additional mechanisms, such as improving learners’ AI literacy and scaffolding the learning processes, may be needed to better motivate learners with both low and advanced proficiency levels for deep engagement. In this sense, proficiency level is not merely a background variable but an active personal factor that configures which AI affordances are available or effective in practice, thereby closing or opening engagement pathways in the AI-mediated environment.

## 5. Conclusions, Limitations, and Future Research Suggestions

This review synthesized 58 empirical studies to examine AI-IDLE through SCT’s person–behavior–environment triad. Speech recognition, natural language processing, and personalized feedback provided opportunities for observation and imitation, while emotional, motivational, and self-belief mechanisms shaped learners’ engagement. These mechanisms could facilitate or hinder learning depending on technological accuracy, human responsiveness, learner goals, and proficiency-task alignment. AI affordances therefore cannot be understood independently of learners’ interpretations and actions. These insights can guide the leveraging of human and AI’s “respective strengths” (Cukurova, 2025, p. 471) to optimize learning outcomes.

Theoretically, this review treats AI affordances as environmental conditions and emotion, motivation, and self-belief as person-level mechanisms connecting those conditions with engagement. This treatment extends SCT to AI-mediated autonomous learning and links AI-IDLE to adaptive intelligence, manifested in learners’ evaluation of AI output, regulation of affect and motivation, calibration of self-perceived competence, and adjustment of behavior to contextual demands. Therefore, although SCT assumes reciprocal influence, the evidence mainly supports environment-to-person-to-behavior pathways; person-to-environment influence remains a potential relationship requiring further study.

Theoretically, this review refines the understanding of technological affordances as an environmental factor and the learners’ interpretation of learning behaviors as the mechanism within AI-IDLE under SCT’s person–behavior–environment triad. Rather than treating AI as a technologically mediated content-delivery system, this study shows that AI technological affordances (e.g., personalized feedback, adaptive content, simulated interactive environments) operate as environmental catalysts that can modulate learners’ personal factors (i.e., emotion, motivation, self-belief) and in turn shape behavioral engagement in informal digital EFL learning. In doing so, the review extends the SCT framework to AI-mediated autonomous digital learning scenarios, where observation and imitation engagements are distributed across human and non-human agents. Moreover, by mapping specific affective and self-belief mechanisms (e.g., anxiety reduction, self-perceived competence) onto AI’s technological affordances, the study offers a more detailed understanding of how learning effectiveness emerges through dynamic interactions between AI tools and the learners themselves in observational and imitative engagement behaviors. We aim to contribute these findings to the ongoing discussion of how SLA happens and operationalizes in AI-rich, out-of-class human–computer interactions.

Practically, the findings suggest several directions for the design and implementation of AI-IDLE. First, learners and educators could intentionally leverage AI technological affordances as the environmental conditions (e.g., low-judgment practice spaces, gamification, nonjudgmental conversational partners) for mechanism interpretations while mitigating the interpretation challenges, such as technological inaccuracies and the lack of human touch through collaborative learning interactions. Second, the insufficiencies found in this study may drive users of AI-learning systems to move beyond an instrumentalist view of AI technologies (Guan & Gu, 2026). Doing so would actively foster critical AI literacy—or lead learners to self-foster their own literacy—so that they could conduct the observational and imitative behaviors needed for learning. Third, given that learners with different levels of language proficiency had concerns about learner–AI interactions, the studies suggest that AI-IDLE intervention designs should be tailored to learners’ profiles to optimize the mechanisms identified in this review for effective learning. Together, these insights can inform more human-centered designs of hybrid learning systems in which AI supports, rather than supplants, human agency.

As a systematic review, this study serves as one of the first steps of AI-integrated educational collaboration. Further studies are encouraged to further explore the interactive dynamics among teachers, learners, and AI under other learning settings (e.g., in-class, out-of-class, interest clubs) with different combinations of technologies (e.g., AI–metaverse and AI–virtual reality).

This review has several limitations that should be acknowledged. First, the corpus was restricted to SSCI-indexed empirical studies published between 2020 and 2025, which may have excluded relevant but non-indexed, earlier, or very recent work, especially in rapidly evolving AI in education contexts. Second, the analysis relied entirely on how primary studies reported AI affordances and interpretations. Observation and imitation were often inferred rather than directly measured; therefore, under-reporting or selective reporting may have biased the thematic patterns. Third, by adopting SCT’s person–behavior–environment triad as the primary analytical lens, we may have foregrounded certain mechanisms (e.g., self-efficacy, anxiety, motivation) while under-emphasizing alternative explanations that could be illuminated by other theoretical perspectives (e.g., ecological, sociomaterial, or critical data studies approaches). Fourth, the included studies involved diverse demographic groups in terms of age, educational level, and language proficiency. Although this diversity facilitated the identification of technological affordances and mechanisms across groups, it limited the generalizability of the findings to any specific demographic group, warranting further research on more homogeneous populations.

Finally, the qualitative synthesis could neither estimate effect sizes nor statistically model relationships among personal, behavioral, and environmental factors. Future research could use broader samples, longitudinal or mixed-methods designs, and meta-analytic approaches to examine how adaptive intelligence and learner self-regulation operate across AI-mediated learning contexts.

## Data Availability

No new data were created or analyzed in this study. Data sharing is not applicable to this article.

## Supplementary Materials

The following supporting information can be downloaded at: https://www.mdpi.com/article/10.3390/jintelligence14080189/s1, The PRISMA checklist; Section S1: The reference list of included studies in the literature review; Section S2: The coding books; Section S3: The included study characteristics for this systematic review.
