# Supplementary material for: Artificial Intelligence in Informal Digital Learning of English: A Systematic Review of Engagement Mechanisms and Learning Processes in Intelligent Learning Systems
Source: J Intell. 2026 Aug 18;14(8):189. doi: 10.3390/jintelligence14080189 (PMC13514965; doi:10.3390/jintelligence14080189)
Supplement: Supplementary file 1 [file jintelligence-14-00189-s001.zip › Supplementary materials.pdf]

Section S1: The reference list of included studies in the literature review

Alrajhi, A. S. (2024). Intelligent personal assistants in self-access L2 vocabulary learning. *Education and Information Technologies*, 1-31. https://doi.org/10.1007/s10639-024-12967-7

Bashori, M., Van Hout, R., Strik, H., & Cucchiarini, C. (2021). Effects of ASR-based websites on EFL learners' vocabulary, speaking anxiety, and language enjoyment. *System*, *99*, 102496. https://doi.org/10.1016/j.system.2021.102496

Biju, N., Abdelrasheed, N. S. G., Bakiyeva, K., Prasad, K. D. V., & Jember, B. (2024). Which one? AI-assisted language assessment or paper format: an exploration of the impacts on foreign language anxiety, learning attitudes, motivation, and writing performance. *Language Testing in Asia*, *14*(45), 1-24. https://doi.org/10.1186/s40468-024-00322-z

Boudouaia, A., Mouas, S., & Kouider, B. (2024). A study on ChatGPT-4 as an innovative approach to enhancing English as a foreign language writing learning. *Journal of Educational Computing Research*, 07356331241247465. https://doi.org/10.1177/07356331241247465

Chen, Y. C. (2024). Effects of technology-enhanced language learning on reducing EFL learners' public speaking anxiety. *Computer Assisted Language Learning*, *37*(4), 789-813. https://doi.org/10.1080/09588221.2022.2055083

Chen, A., Jia, J., Li, Y., & Fu, L. (2024). Investigating the Effect of Role-Play Activity With GenAI Agent on EFL Students' Speaking Performance. *Journal of Educational Computing Research*, 07356331241299058. https://doi.org/10.1177/0735633124129905

Chen, H. H. J., Yang, C. T. Y., & Lai, K. K. W. (2023). Investigating college EFL learners' perceptions toward the use of Google Assistant for foreign language learning. *Interactive Learning Environments*, *31*(3), 1335-1350. https://doi.org/10.1080/10494820.2020.1833043

Cui, W., & Zhang, J. (2025). AI applications in language learning: improving speaking fluency and pronunciation using chatbots and speech feedback. *Interactive Learning Environments*, 1–16. https://doi.org/10.1080/10494820.2025.2546634

Ebadi, S., & Amini, A. (2024). Examining the roles of social presence and human-likeness on Iranian EFL learners' motivation using artificial intelligence technology: A case of CSIEC chatbot. *Interactive Learning Environments*, *32*(2), 655-673. https://doi.org/10.1080/10494820.2022.2096638

Fathi, J., & Rahimi, M. (2024). Utilising artificial intelligence-enhanced writing mediation to develop academic writing skills in EFL learners: a qualitative study. *Computer Assisted Language Learning*, 1-40. https://doi.org/10.1080/09588221.2024.2374772

Fathi, J., Rahimi, M., & Derakhshan, A. (2024). Improving EFL learners' speaking skills and willingness to communicate via artificial intelligence-mediated interactions. *System*, *121*, 103254. https://doi.org/10.1016/j.system.2024.103254

Feng, L. (2024). Investigating the Effects of Artificial Intelligence-Assisted Language

Learning Strategies on Cognitive Load and Learning Outcomes: A Comparative Study. *Journal of Educational Computing Research*, 07356331241268349. https://doi.org/10.1177/07356331241268349

Feng, Y., & Wang, X. (2023). A comparative study on the development of Chinese and English abilities of Chinese primary school students through two bilingual reading modes: human-AI robot interaction and paper books. *Frontiers in Psychology*, *14*, 1200675. https://doi.org/10.3389/fpsyg.2023.1200675

Guo, Y., & Wang, Y. (2024). Exploring the Effects of Artificial Intelligence Application on EFL Students' Academic Engagement and Emotional Experiences: A Mixed-Methods Study. *European Journal of Education*, e12812. https://doi.org/10.1111/ejed.12812

Guan, L., Zhang, E. Y., & Gu, M. M. (2025). Examining generative AI–mediated informal digital learning of English practices with social cognitive theory: A mixed-methods study. *ReCALL*, *37*(3), 315-331. doi:10.1017/S0958344024000259

Hsu, H. L., & Chen, H. J. H. (2025). Examining the impact of Intelligent Personal Assistants with self-reflection on language learning beyond the classroom. *Computer Assisted Language Learning*, 1–27. https://doi.org/10.1080/09588221.2025.2506478

Hsu, H. L., Chen, H. H. J., & Todd, A. G. (2023). Investigating the impact of the Amazon Alexa on the development of L2 listening and speaking skills. *Interactive Learning Environments*, *31*(9), 5732-5745. https://doi.org/10.1080/10494820.2021.2016864

Hong, X., Guo, L. (2025).Effects of AI-enhanced multi-display language teaching systems on learning motivation, cognitive load management, and learner autonomy. *Education and Information Technologies, 30*, 17155–17189. https://doi.org/10.1007/s10639-025-13472-1

Hwang, W. Y., Nurtantyana, R., Purba, S. W. D., Hariyanti, U., Indrihapsari, Y., & Surjono, H. D. (2023). AI and recognition technologies to facilitate English as foreign language writing for supporting personalization and contextualization in authentic contexts. *Journal of Educational Computing Research*, *61*(5), 1008-1035. https://doi.org/10.1177/07356331221137253

Hajmalek, M. M., & Sabouri, S. (2025). Tapping into second language learners' musical intelligence to tune up for computer-assisted pronunciation training. *Computer Assisted Language Learning*, 1–23. https://doi.org/10.1080/09588221.2025.2488855

Jiang. R., Yang. G., & Shen. Q. (2025), The Motivational Impact of GenAI Tools in Language Learning: a Quasi-Experiment Study. *International Journal of Applied Linguistics, 35*, 1338-1350. https://doi.org/10.1111/ijal.12701

Jeon, J., & Lee, S. (2024). The impact of a chatbot-assisted flipped approach on EFL learner interaction. *Educational Technology & Society*, *27*(4), 218-234. https://doi.org/10.30191/ETS.202410_27(4).RP12

Khodabandeh, F. (2025). The impact of AI-Assisted vs. instructor-provided feedback on Awe, autonomy, and letter writing skills in introverted and extroverted EFL

learners in online learning environments. *Journal of Educational Computing Research, 64*(3), 658-691. https://doi.org/10.1177/07356331251412228

Karataş, F., Abedi, F. Y., Ozek Gunyel, F., Karadeniz, D., & Kuzgun, Y. (2024). Incorporating AI in foreign language education: An investigation into ChatGPT's effect on foreign language learners. *Education and Information Technologies*, *29*. 1-24. https://doi.org/10.1007/s10639-024-12574-6

Liu, G. L., Darvin, R., & Ma, C. (2024). Exploring AI-mediated informal digital learning of English (AI-IDLE): a mixed-method investigation of Chinese EFL learners' AI adoption and experiences. *Computer Assisted Language Learning*, 1-29. https://doi.org/10.1080/09588221.2024.2310288

Liu, C., Hou, J., Tu, Y. F., Wang, Y., & Hwang, G. J. (2023). Incorporating a reflective thinking promoting mechanism into artificial intelligence-supported English writing environments. *Interactive Learning Environments*, *31*(9), 5614-5632. https://doi.org/10.1080/10494820.2021.2012812

Liu, C. C., Liao, M. G., Chang, C. H., & Lin, H. M. (2022). An analysis of children's interaction with an AI chatbot and its impact on their interest in reading. *Computers & Education*, *189*, 104576. https://doi.org/10.1016/j.compedu.2022.104576

Qiao, H., & Zhao, A. (2023). Artificial intelligence-based language learning: illuminating the impact on speaking skills and self-regulation in Chinese EFL context. *Frontiers in Psychology*, *14*, 1255594. https://doi.org/10.3389/fpsyg.2023.1255594

Rahman, A., & Tomy, P. (2024). Intelligent personal assistant-An interlocutor to mollify foreign language speaking anxiety. *Interactive Learning Environments*, *32*(8), 4569-4586. https://doi.org/10.1080/10494820.2023.2204324

Sasaki, M., Mizumoto, A., & Matsuda, P. K. (2024). Machine translation as a form of feedback on L2 writing. *International Review of Applied Linguistics in Language Teaching*, https://doi.org/10.1515/iral-2023-0223.

Shafiee Rad, H. (2024). Revolutionizing L2 speaking proficiency, willingness to communicate, and perceptions through artificial intelligence: a case of Speeko application. *Innovation in Language Learning and Teaching, 18*(4), 364–379. https://doi.org/10.1080/17501229.2024.2309539

Song, C., & Song, Y. (2023). Enhancing academic writing skills and motivation: assessing the efficacy of ChatGPT in AI-assisted language learning for EFL students. *Frontiers in Psychology*, *14*, 1260843. https://doi.org/10.3389/fpsyg.2023.1260843

Tai, T. Y. (2024). Effects of intelligent personal assistants on EFL learners' oral proficiency outside the classroom. *Computer Assisted Language Learning*, *37*(5-6), 1281-1310. https://doi.org/10.1080/09588221.2022.2075013

Tai, T. Y., & Chen, H. H. J. (2024a). Improving elementary EFL speaking skills with generative AI chatbots: Exploring individual and paired interactions. *Computers & Education*, *220*, 105112. https://doi.org/10.1016/j.compedu.2024.105112

Tai, T. Y., & Chen, H. H. J. (2024b). The impact of intelligent personal assistants on

adolescent EFL learners' listening comprehension. *Computer Assisted Language Learning*, *37*(3), 433-460. https://doi.org/10.1080/09588221.2022.2040536
Tai, T. Y., & Chen, H. H. J. (2023). The impact of Google Assistant on adolescent EFL learners' willingness to communicate. *Interactive Learning Environments*, *31*(3), 1485-1502. https://doi.org/10.1080/10494820.2020.1841801
Wang, D. (2024a). Teacher-versus AI-generated (Poe application) corrective feedback and language learners' writing anxiety, complexity, fluency, and accuracy. *International Review of Research in Open and Distributed Learning*, *25*(3), 37-56. https://doi.org/10.19173/irrodl.v25i3.7646
Wang, Y. (2024b). Cognitive and sociocultural dynamics of self-regulated use of machine translation and generative AI tools in academic EFL writing. *System*, *126*, 103505. https://doi.org/10.1016/j.system.2024.103505
Wu, X., Li, R., & Gwendoline Quek, C. L. (2025). When GenAI chatbots meet self-determination: unpacking Chinese EFL learners' engagement in chatbot-assisted language learning. *Interactive Learning Environments*, 1–21. https://doi.org/10.1080/10494820.2025.2538756
Wang, C., Zou, B., Du, Y., & Wang, Z. (2024). The Impact of Different Conversational Generative AI Chatbots on EFL Learners: an Analysis of Willingness to Communicate, Foreign Language Speaking Anxiety, and Self-perceived Communicative Competence. *System*, 103533. https://doi.org/10.1016/j.system.2024.103533
Wei, L. (2023). Artificial intelligence in language instruction: impact on English learning achievement, L2 motivation, and self-regulated learning. *Frontiers in Psychology*, *14*, 1261955. https://doi.org/10.3389/fpsyg.2023.1261955
Woo, D. J., Wang, D., Guo, K., & Susanto, H. (2024). Teaching EFL students to write with ChatGPT: Students' motivation to learn, cognitive load, and satisfaction with the learning process. *Education and Information Technologies*, 1-28. https://doi.org/10.1007/s10639-024-12819-4
Yan, D. (2023). Impact of ChatGPT on learners in a L2 writing practicum: An exploratory investigation. *Education and Information Technologies, 28*, 13943–13967. https://doi.org/10.1007/s10639-023-11742-4
Yan, D., & Zhang, S. (2024). L2 writer engagement with automated written corrective feedback provided by ChatGPT: A mixed-method multiple case study. *Humanities and Social Sciences Communications*, *11*(1), 1-14. https://doi.org/10.1057/s41599-024-03543-y
Yuan, Y. (2024). An empirical study of the efficacy of AI chatbots for English as a foreign language learning in primary education. *Interactive Learning Environments*, 1-16. https://doi.org/10.1080/10494820.2023.2282112
Yuan, L., & Liu, X. (2025). The effect of artificial intelligence tools on EFL learners' engagement, enjoyment, and motivation. *Computers in Human Behavior*, *162*, 108474. https://doi.org/10.1016/j.chb.2024.108474
Yang, Y., & Chen, L. (2025). Beyond concordances: exploring GenAI-assisted data-driven learning for English periphrastic causative constructions from a sociocultural perspective. *Computer Assisted Language Learning*, 1–31.

https://doi.org/10.1080/09588221.2025.2513988

Yang, H., Kim, H., Lee, J. H., & Shin, D. (2022). Implementation of an AI chatbot as an English conversation partner in EFL speaking classes. *ReCALL*, *34*(3), 327-343. doi:10.1017/S0958344022000039

Yang, C. T. Y., Lai, S. L., & Chen, H. H. J. (2024). The impact of intelligent personal assistants on learners' autonomous learning of second language listening and speaking. *Interactive Learning Environments*, *32*(5), 2175-2195. https://doi.org/10.1080/10494820.2022.2141266

Zhang, M. (2024). Enhancing self-regulation and learner engagement in L2 speaking: exploring the potential of intelligent personal assistants within a learning-oriented feedback framework. *BMC psychology*, *12*(1), 421. https://doi.org/10.1186/s40359-024-01917-0

Zhang, Z. (2025). The Role of Artificial Intelligence Tools on Chinese EFL Learners' Self-Regulation, Resilience and Autonomy. *European Journal of Education, 60*(2), 1-12. https://doi.org/10.1111/ejed.70127

Zhang, C., Meng, Y., & Ma, X. (2024). Artificial intelligence in EFL speaking: Impact on enjoyment, anxiety, and willingness to communicate. *System*, *121*, 103259. https://doi.org/10.1016/j.system.2024.103259

Zhou, C., & Hou, F. (2024). Can AI Empower L2 Education? Exploring Its Influence on the Behavioural, Cognitive and Emotional Engagement of EFL Teachers and Language Learners. *European Journal of Education*, e12750. https://doi.org/10.1111/ejed.12750

Zhou, Q., Hashim, H., & Sulaiman, N. A. (2025). Integrating AI chatbots in informal digital English learning: Impacts on listening competencies in Chinese higher education. *Education and Information Technologies, 30*, 27031-27059. https://doi.org/10.1007/s10639-025-13811-2

Zou, B., Du, Y., Wang, Z., Chen, J., & Zhang, W. (2023). An investigation into artificial intelligence speech evaluation programs with automatic feedback for developing EFL learners' speaking skills. *Sage Open*, *13*(3), 21582440231193818. https://doi.org/10.1177/21582440231193818

Zou, B., Lyu, Q., Han, Y., Li, Z., & Zhang, W. (2025a). Exploring students' acceptance of an artificial intelligence speech evaluation program for EFL speaking practice: an application of the Integrated Model of Technology Acceptance. Computer Assisted Language Learning, 38(5–6), 1366–1391. https://doi.org/10.1080/09588221.2023.2278608

Zou, B., Liviero, S., Ma, Q., Zhang, W., Du, Y., & Xing, P. (2024). Exploring EFL learners' perceived promise and limitations of using an artificial intelligence speech evaluation system for speaking practice. *System*, *126*, 103497. https://doi.org/10.1016/j.system.2024.103497

Zou, M., Soyoof, A., & Amjad, F. (2025b). Bridging the Digital Divide: Multilingual Young People's Growth Mindset and Emotions in Artificial Intelligence-Mediated Informal Digital Learning of English Engagement. *RELC Journal*, 00336882251372643. https://doi.org/10.1177/00336882251372643

Section S2: ***The coding books***

For each included study, we extracted full-sentence excerpts that described AI technological affordances and/or learner-side mechanisms of action. We then assigned one or more codes to each excerpt. Codes were not mutually exclusive: a single excerpt could receive multiple codes if it met the inclusion criteria for more than one category. For reporting purposes in the main text and appendices, all frequency counts (n) refer to the number of studies in which a given code appeared at least once, rather than to the raw number of coded excerpts. The tables below summarize the main code categories, their sub-themes, and brief operational descriptions, together with a sample quote for illustration. A study-by-code matrix (Study*technology/uses) is provided in Section S3 for coding verifications.

**AI's technological affordances**		
**Themes**	**Sub-themes**	**Sample quotes**
In speaking studies	Speech recognition	"... automated speech recognition technology to evaluate their performance..."
	Natural language processing	"... to administer personalized and interactive English language instruction..."
	Personalized feedback	"... specifically designed to provide corrective feedback..."
	Gamification	"... game-like or fun features..."
	Searching and providing information	"... research-based tips on overcoming speech anxiety..."
	Translation	"...English-to-Chinese translation support"
In writing studies	Personalized feedback	"... personalized scoring feedback..."
	Translation	"... translation between languages"
	Multimodality	"... providing multimodality inputs..."
In reading studies	Natural language processing	"... simply texts so that students can read the original text and the ChatGPT-simplified text..."
In listening studies	Translation	"... Youdao translator allows me to translate during fully English-proficient courses..."
In other EFL studies (e.g., engagement, affection)	Gamification	"... employs educational games..."
	Assessment	"... collects online classroom data to support formative assessment..."
	Personalized feedback	"... personalized learning experiences and adaptive feedback provided by AI..."

AI's interpretation mechanisms (positive)		
**Theme**	**Sub-theme**	**Sample Quote**
Emotion	Lowered anxiety	"clear drop in anxiety levels..."
	Heightened enjoyment	"... increased foreign language enjoyment./
	Comfort and relaxing	"...feel comfortable when talking with the chatbot..."
	Fun and interesting	"... didn't feel like learning but like playing games..."
	Feeling novel	"... due to "novelty effect" of tech no logy..."
Motivation	Useful	"... useful in coping with challenging reading tasks..."
	Convenience	"... a convenient language learning tool that..."
	More opportunities for practice	"... the unique opportunity presented by ChatGPT..."
	Autonomy	"... having self-control in learning pace..."
	Human-like interactions	"…AI is human-like..."
	Language quality	"AI provides more authentic English than teachers."
Self-belief	Self-believes in language compete nee	"... higher self-perceived competence..."
	Self-believes in language compete nee development	"... clear development of writing abilities..."

AI's interpretation mechanisms (negative)		
**Theme**	**Sub-theme**	**Sample Quote**
Emotion	Technological reasons	"... inability to record users' improvement produced unpleasant feelings..."
	Pedagogical reasons	"... but still anxious while talking with people..."
Motivation	Technological reasons	"... there are accent recognition errors..."
	Pedagogical reasons	"... teachers were not able to assist..."

Self-belief	Proficiency reasons	"... difficult to understand some expressions in the chatbots speech..."

Section S3: The included study characteristics for this systematic review

Authors(year)	Technology	Technology uses	Sample Size	Main Learning Outcomes
Zhang et al. (2024)	an AI-speaking assistant	Additional learning opportunities	131	Speaking
Bashori et al. (2021)	Automatic speech recognition	Speech recognition	232	Speaking
Rahman & Tomy (2024)	Google Assistant	AI interactive partner, additional learning opportunities	86	Speaking
Woo et al. (2024)	ChatGPT	Personalized feedback	21	Writing
Tai & Chen (2024b)	Google Home Hub	AI interactive partner	88	Speaking
Tai (2024)	Google Assistant	Multimodal feedback	89	Speaking
Biju et al. (2024)	ChatGPT	Personalized feedback	70	Writing
Tai & Chen (2024a)	CoolE bot	AI interactive partner	85	Speaking
Wei (2023)	Duolingo	AI interactive partner, personalized feedback	14	Speaking
Chen et al. (2023)	Google assistant	AI interactive partner, additional learning opportunities	29	Speaking
Tai & Chen (2023)	Google assistant	AI interactive partner	112	Speaking
Yang et al. (2022)	Ellie	AI interactive partner, additional learning opportunities	314	Speaking
Feng & Wang (2023)	AI robots (unspecified)	AI as the source of learning materials	20	Reading
Liu et al. (2022)	Sister Fish	AI interactive partner	68	Reading
Fathi & Rahimi (2024)	ChatGPT	Personalized feedback	14	Writing

Fathi et al. (2024)	Andy English Chatbot	AI interactive partner	65	Speaking
Chen (2024)	Orai	AI interactive partner, personalized feedback	33	Speaking
Liu et al. (2023)	Mosoteach	Extra scaffolding	20	Writing
Ebadi & Amini (2024)	CSIEC	AI interactive partner	256	Learning behaviors
Song & Song (2023)	ChatGPT	AI interactive partner, personalized feedback	50	Writing
Yuan (2024)	Mondly	Authentic practice opportunities	74	Speaking
Guan et al. (2025)	IFlyTech	AI interactive partner, personalized feedback	47	Speaking
Guo & Wang (2024)	Bodoudou	Educational games	10	Engagement
Zhou & Hou (2024)	Various AI tools	Information searching; feedback; conversation; idea generation	38	Engagement
Wang et al. (2024)	Various AI tools	Additional learning opportunities	33	Speaking
Liu et al. (2024)	Various AI tools	Self-regulated usage of AI	867	Perceived emotions, regulation strategies
Wang (2024b)	ChatGPT, Google Translate, DeepL	Personalized feedback, translation	79	Writing
Boudouaia et al. (2024)	ChatGPT	Personalized feedback,	76	Writing
Zhang (2024)	Google Assistant	Additional learning opportunities	54	Speaking
Alrajhi (2024)	Google Assistant	Translation	74	Vocabulary
Karataş et al. (2024)	ChatGPT	AI-based assessment, feedback, interaction	13	Writing
Hwang et al. (2023)	Smart RoamLingo	Additional learning opportunities, personalized feedback	104	Writing
Yan (2023)	ChatGPT	Brainstorming, additional learning opportunities, personalized feedback	35	Writing
Qiao & Zhao	Duolingo	Additional learning	93	Speaking

(2023)		opportunities, personalized feedback		
Zou et al. (2024)	EAP Talk	Personalized feedback	366	Speaking
Jeon & Lee (2024)	Dialogflow	Personalized feedback	87	Speaking
Feng (2024)	Not specified	Additional learning opportunities, personalized feedback, speech recognition	484	Listening, reading
Zou et al. (2025a)	EAP Talk	AI-based assessment, feedback	218	Speaking
Yuan & Liu, (2025)	Duolingo	AI interactive partner, additional learning opportunities	412	Engagement, enjoyment, motivation
Yan & Zhang (2024)	ChatGPT	Personalized feedback	4	Writing
Zou et al. (2023)	Various AI tools	Personalized feedback	40	Speaking
Wang (2024a)	POE	Personalized feedback	75	Writing
Shafiee Rad (2024)	Speeko	AI interactive partner, personalized feedback, speech recognition	66	Speaking
Sasaki et al. (2024)	DeepL	Translation	23	Writing
Chen et al. (2024)	Not specified	AI interactive partner	53	Speaking
Yang et al. (2024)	Google Assistant	AI interactive partner, additional learning opportunities, gaming	34	Speaking, listening
Zhang (2025)	ERNIE Bot, Kimi Chat, SpeakGuru	AI interactive partner	94	Regulation
Zhou et al. (2025)	ChatGPT	AI interactive partner	67	Listening
Jiang et al. (2025)	Not specified	AI interactive partner, additional learning opportunities	355	Motivation
Hsu & Chen (2025)	Amazon smart speakers, Alexa	AI interactive partner	33	Speaking, listening
Hong & Guo (2025)	Self-designed AI-VR system	AI interactive partner	302	Motivation, engagement
Khodabandeh	ChatGPT	Personalized feedback	130	Writing

(2025)				
Hajmalek & Sabouri (2025)	Adobe Audition, Ableton Live	Automatic Speech Recognition	39	Speaking
Cui & Zhang (2025)	Andy chatbot, Speechace	Personalized feedback	60	Speaking
Yang & Chen (2025)	Not specified	AI interactive partner	40	Learning perception
Zou et al. (2025b)	Various AI tools	AI interactive partner	369	Engagement, emotions
Wu et al. (2025)	Various AI tools	AI interactive partner	506	Engagement
Hsu et al. (2023)	Alexa	AI interactive partner	50	Speaking, listening
